# Hand hygiene campaigns in a low resource context: a Vietnam perspective

**DOI:** 10.1186/1753-6561-5-S6-O22

**Published:** 2011-06-29

**Authors:** S Salmon, VH Nguyen, M-L McLaws, D Pittet, C Kilpatrick, TAT Le, AT Truong

**Affiliations:** 1 School of Public Health & Community Medicine, University of New South Wales, Australia, Kensington, NSW, Australia; 2 Infection Control, Bach Mai Hospital, Hanoi, Vietnam; 3 Infection Control Programme and, University of Geneva Hospitals and Faculty of Medicine, Geneva, Switzerland; 4 WHO Global Alliance on Patient Safety, World Health Organisation, Geneva, Switzerland; 5 Infection Control, Cho Ray Hospital, Ho Chi Minh City, Vietnam

## Introduction / objectives

Bach Mai tertiary hospital is a 1900 bed facility in Viet Nam. Previous hospital hand hygiene programs proved unsuccessful which prompted the director to launch an intensive hand hygiene campaign on 5^th^ May 2009 to reduce health-care associated infection (HAI) using World Health Organization (WHO) tools. We would like to present the results and challenges of the Bach Mai hospital hand hygiene campaign.

## Methods

A review of hand hygiene compliance rates before and after a two-month campaign. The campaign launched a practical hand hygiene protocol including provision of soap/water and alcohol-based hand rub (ABHR); education and communication materials; and a 20-hour hand hygiene training course for link nurses. Daily audits were done by accredited link nurses in 29 clinical departments. Compliance data was analyzed and results distributed.

## Results

In 2007, 2526 hand hygiene observations showed compliance rates of 14.0% (95%CI 12.7%>15.5%). After the 2009 campaign the rate improved significantly (p<0.0001) to 47.0% (1806/3840) (95%CI 45.4%>48.6%). Factors impeding compliance included inappropriate glove use and access to soap/water and ABHR.

## Conclusion

The campaign improved compliance by three-fold, however compliance remains less than optimal. Commitment to improving compliance is needed from hospital leadership levels. Current research conducted by the University of New South Wales, Bach Hospital Hanoi and WHO aims to improve hand hygiene and reduce HAIs using standardised surveillance tools. We believe that this will provide evidence of the impacts of hand hygiene on patient safety in a sample of health care facilities in Viet Nam.

## Disclosure of interest

None declared.

